# Graphene Oxide-Based Targeting of Extracellular Cathepsin D and Cathepsin L As A Novel Anti-Metastatic Enzyme Cancer Therapy

**DOI:** 10.3390/cancers11030319

**Published:** 2019-03-06

**Authors:** Tanveer A. Tabish, Md Zahidul I. Pranjol, David W. Horsell, Alma A. M. Rahat, Jacqueline L. Whatmore, Paul G. Winyard, Shaowei Zhang

**Affiliations:** 1 College of Engineering, Mathematics and Physical Sciences, University of Exeter, Exeter EX4 4QF, UK; D.W.Horsell@exeter.ac.uk (D.W.H.); A.A.M.Rahat@exeter.ac.uk (A.A.M.R.); 2 Institute of Biomedical and Clinical Science, University of Exeter Medical School, St Luke’s Campus, Exeter EX1 2LU, UK; z.pranjol@qmul.ac.uk; 3 William Harvey Research Institute, Barts and the London School of Medicine and Dentistry, Queen Mary University of London, London EC1M 6BQ, UK; 4 School of Computing, Electronics and Mathematics, University of Plymouth, Plymouth PL4 8AA, UK

**Keywords:** graphene oxide, adsorption, cathepsin D, cathepsin L, anti-metastatic enzyme cancer therapy

## Abstract

Overexpression and secretion of the enzymes cathepsin D (CathD) and cathepsin L (CathL) is associated with metastasis in several human cancers. As a superfamily, extracellularly, these proteins may act within the tumor microenvironment to drive cancer progression, proliferation, invasion and metastasis. Therefore, it is important to discover novel therapeutic treatment strategies to target CathD and CathL and potentially impede metastasis. Graphene oxide (GO) could form the basis of such a strategy by acting as an adsorbent for pro-metastatic enzymes. Here, we have conducted research into the potential of targeted anti-metastatic therapy using GO to adsorb these pro-tumorigenic enzymes. Binding of CathD/L to GO revealed that CathD/L were adsorbed onto the surface of GO through its cationic and hydrophilic residues. This work could provide a roadmap for the rational integration of CathD/L-targeting agents into clinical settings.

## 1. Introduction

Every year more than 2.28 million new cases of breast and ovarian cancers are diagnosed worldwide, principally in developed countries and 807,440 women die of them [1,2,3], with these cancers representing the first and fifth most common cause of female malignancies, respectively [4,5]. Although these diseases have different pathologies they share a common set of molecular mechanisms such as the misfolding/aggregation, overexpression and hypersecretion of specific proteins typically involved in degrading cross-linked, abnormal, short-lived self- and foreign- proteins in lysosomes and phagocytosis. The intracellular and extracellular responses of the tumor microenvironment tend to be more prominent in response to conditions such as acidic pH [6], the enhanced permeability and retention effect [7], the enzyme abundance in the tumor extracellular matrix, [8] and overexpression of particular cell membrane receptors [9]. Typically, this emanates from the misfolding of proteins which potentially tend to form pathogenic aggregates, including harmful oligomeric and/or cytotoxic factors involved in the molecular etiology of these diseases and other pathologies (which are linked with the ability of the proteins to fully execute their physiological functions provided by certain regions of their protein sequence) [10]. When the intracellular protein degradation within the cells’ acidic endosomal/lysosome compartments increases, the proteolytic activity becomes particularly high in lysosomal proteases such as cathepsin D (CathD) and cathepsin L (CathL). However, in tumor invasion and development, these enzymes play a significant role by extracellularly influencing cell proliferation, differentiation, cell migration, programmed cell death, angiogenesis, immune defence, inflammation and extracellular tissue remodelling [11,12].

Higher CathL and CathD concentrations are closely related to an increased risk of metastasis [13]. For example, CathL is considered to be associated with tumor invasion and metastasis, by degrading subunits of extracellular matrix including proteoglycans, elastin, entactin (nidogen), laminin, fibronectin, perlecan and interstitial and basement-membrane collagens. Recently, we showed a significantly higher expression of CathL in the omentum hosting metastatic ovarian serous carcinoma compared with omentum from normal and benign controls with ovarian cystadenoma. We found that exogenous CathL induced pro-angiogenic effects on omental microvascular endothelial cells which may aid metastasis [14]. Recent studies have investigated the enhanced immunohistochemical CathD expression as an indicator of potential malignancy in serous ovarian cancer [13]. For example Losch et al. [15] demonstrated that CathD was detected in more than 70% of invasive ovarian cancers. Secreted CathD from breast cancer cells and its proteolytic role in degrading ECM proteins and subsequently releasing growth factors such as bFGF, have also been reported, which provide an ability for cancer cells to invade nearby tissue [16,17]. Misfolding, overexpression and hypersecretion of CathD and CathL have now been demonstrated in numerous cancer types such as ovarian, breast, lung and prostate, endometrial, as well as malignant glioma and melanoma and are recognized as critical players in cancer biology by regulating diverse proteolytic functions in triggering the breakdown of the tumor basement membrane and fueling tumor invasion [18,19,20,21]. Adsorption of these enzymes to two-dimensional materials opens a window of opportunity to develop a wide range of new approaches in the prevention of cancer.

Nanotechnology and its underpinning sciences have significantly contributed to the improvement of nanodrug bioavailability and therapeutic index in cancer therapy [22]. Recently, graphene oxide (GO) formulations have been developed into adaptable nanoscale platforms for medical interventions as one of the most sophisticated and minimally toxic tools [23] that permit direct contact with, and manipulation of, the intracellular environment. Graphene is a two-dimensional sheet composed of a single layer of *sp*^2^-hybridized carbon atoms arranged in a honeycomb lattice [24]. Graphene and its analogues have attracted tremendous interest over the last decade for use in biomedicine owing to their unique physicochemical and mechanical properties, interesting optical and electronic properties, large surface area and good biofunctionality [25]. GO has many advantages over conventional nanosheets and other derivatives of graphene, such as a small size, chemical inertness, high specific surface area, photo-stability, good water solubility, high drug loading capacity, high purity, good fluorescence capability and biocompatibility. These properties make GO a promising candidate in novel delivery systems for target-specific therapeutic drugs and for the diagnosis of different medical conditions as well as for wound healing [26,27]. Furthermore, GO could offer a potential therapeutic tool by adsorbing the pro-metastatic enzymes, which are cancer-associated factors. GO has a large interfacial area and spatial constraints for biological interaction, ideally suited to constructing a robust and cost-effective extracellular tumor-specific enzyme binding method [28]. This capability of GO to bind and track an active enzyme could open the door to new clinical algorithms based on ‘enzyme-targeted therapy’. GO nanoformulations which take up these enzymes could be key enablers of novel anti-metastatic enzyme therapy by breaking down the functional and structural integrity of extracellular enzymes. These GO nanoplatfoms offer a simple, safe and robust strategy in boosting the concept of ‘anti-metastatic enzyme-targeted therapy’, a neologism coined to indicate an innovative and revolutionary approach useful to adsorb and treat ‘pro-tumorigenic’ enzymes with a number of outcomes: notably the clearance of these enzymes, their structural breakdown, their digestion to active site-directed specific adsorbents and the deregulation of pro-tumorigenic enzymes. It is generally understood that the biocompatibility of graphene-based materials is limited by their sharp edges and two-dimensional monolayered structures, which is evident from concentration-dependent toxic effects in numerous cell lines. Targeting and therapeutic adsorption of CathD and CathL in cancer treatment are currently unknown and undefined. The process of enzyme adsorption, and its therapeutic efficacy are affected by several factors such as: the properties of proteins and their concentrations in solution; pH and ionic strength; the temperature of the medium; pH-dependent adsorption performance; the structural stability of proteins; the selection and nature of adsorbent, porous sites/vacancies in adsorbents to take up the proteins; and strength/stability of adsorbate-adsorbent interface. The mechanistic aspects of protein adsorption and/or protein corona formation as a result of the interaction of proteins with graphene may involve electrostatic and hydrophobic interactions [29]. The intrinsic stability of the adsorbent matrix structure, which can be revealed by undergoing structural rearrangements, and conformational alterations, resulting in protein denaturation and/or loss of functional activities and a change in surface energy, allows a wide range of chemical changes in functional groups and wettabilities. The established method of fluorescence quenching and absorbance, together with vibrational spectrometry, wetting transparency, adsorption kinetics, and regression analysis can be used to reveal the fundamental aspects of the enzyme-graphene interaction and to address a variety of pre-clinical unknowns in the same theranostic session.

**Figure 1:**
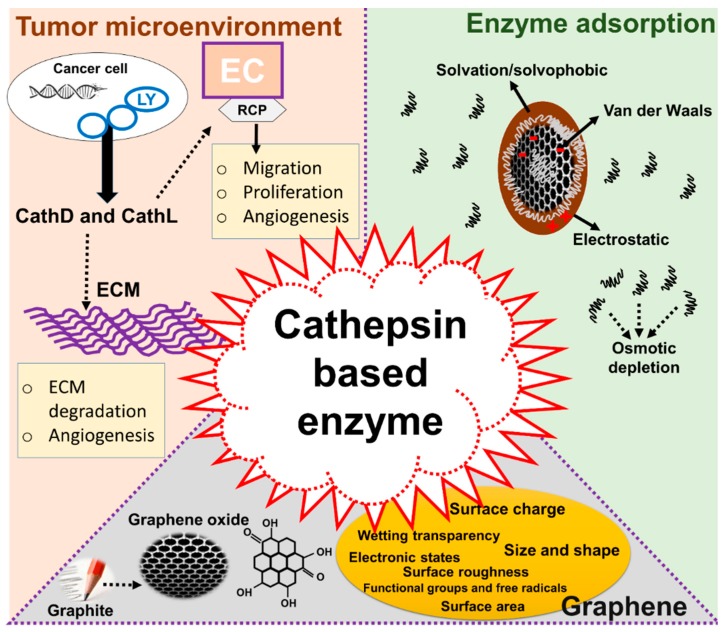
Proposed mechanism of cathepsin function in cancer metastasis and use of graphene oxide (GO) as an adsorbent to remove cathepsin from a living system. There are three panels in this diagram: (1) role of cathepsins in cancer progression: (2) structure of GO and its parameters relevant to the adsorption of cathepsins: and (3) the mechanism of adsorption. The left panel (1) illustrates possible tumorigenic and proangiogenic roles of cathepsin D (CathD) and cathepsin L (CathL) within the cancerous stroma or extracellular matrix (ECM) on endothelial cells (EC) acting via an unknown receptor(s) (RCP). The bottom panel (2) shows the structure of GO. This is prepared from graphite using the modified Hummer’s method [26,27,28]. GO has suitable properties for the efficient adsorption of these enzymes such as surface charge, surface area, functional groups, electronic and chemical properties. The right panel (3) shows the potential mechanism involved in enzyme internalization, the interaction of GO with CathD/CathL and the further breakdown of cathepsins which may lead to cathepsin removal. Electrostatic and van der Waals forces, osmotic depletion and solvophobicity play a pivotal role in adsorption of such enzymes.

Secretion of CathD and CathL poses a unique therapeutic challenge in breast and ovarian cancers. Therefore, a fuller clearance of these proteins before their involvement in secondary tumour progression may help advancement of treatment modalities. We have previously reported on the expression and secretion of CathL and CathD in the omentum and ascites of ovarian malignant patients, as well as in the tumor-based conditioned media ovarian cancer cell lines [14]. In the present paper, we report the use of GO to investigate whether CathD and CathL might be cleared out through an adsorption process. To help visualize the role that GO plays, we used a cost-effective and scalable batch adsorption approach, where complementary information is channeled via multimodal kinetic and regression models as an analogy of a multiplexed toxicity-dependent clearance of pro-metastasis enzymes. Our study reveals that inhibition of CathD and CathL could indeed help overcome the therapeutic challenges faced in breast and ovarian cancers. The idea of enzyme-targeting therapy is explained in Figure 1.

## 2. Results

### 2.1. Synthesis and In Vitro Toxic Effects of GO on Lung Cancer Cells

Similar to our previously reported work, exfoliated GO was synthesized following the modified Hummer’s method [30,31,32]. The basic characterization is given in Supplementary Note 1 and Figures S1–S9. TEM imaging showed the flake-like shapes of GO (Figure S1). We first characterized GO, referred to as GO sheets, indicating atomic compositions of C (1s) and O(1s) as 91%, and 9% (Figure S2A), respectively. The binding energy of 285.0 eV was related to the C−C, C=C, and C−H bonds (Figure S2B). The other C_1s_ peaks of GO contained three main components belonging to C−O (hydroxyl and epoxy, 286.7 eV), C=C/C−C (284.7 eV) and O=C−O (carboxyl, 288.8 eV) and a minor component of the C=O (carbonyl, 287.4 eV) and O=C−OH (289.1 eV) functional groups [33]. The Raman spectrum of GO (Figure S3) exhibited a D band at 1358 cm^−1^ (the presence of defects) and a G band at 1595 cm^−1^ (the in-plane stretching motion of pairs of sp^2^ atoms) [34]. The surface area of the GO was measured by the N_2_ absorption Brunauer−Emmett−Teller (BET) method and found to be 25 m^2^/g with a pore volume of 0.07 cm^3^/g (Figure S4). The surface charge of the GO sheets was determined by the zeta potential measurements (Figure S5). The GO sheets were highly negatively charged (−63.54 mV) due to the presence in their molecular structure of the carboxyl group in the free state. Furthermore, the FTIR spectrum of GO (Figure S6) showed the specific functional groups of C−O−C (~1000 cm^−1^), C−O (1230 cm^−1^), C=C (~1620 cm^−1^) and C=O (1740–1720 cm^−1^) bonds. The band in the region of 3600–3300 cm^−1^ corresponds to O−H stretching vibrations of hydroxyl and carboxyl groups of GO [35]. The Lambert–Beer law, which describes the linear relationship between the absorbance and the concentration of the compound in a given solution was used to examine the dispersibility of GO. A calibration curve was constructed by measuring the absorbance at 232 nm of nine different concentrations (0.039–10 mg/mL) of the GO solution, in which there was good water dispersibility of GO (Figure S7) [36]. The XRD pattern of GO, as prepared in the present study, gave a (001) reflection peak at 2*θ =* 13.7° (Figure S8), which corresponds to a d-spacing of 0.75 nm, and exhibits an increased interlayer distance compared to that (3.34 Å) (2 theta 1/4 26.7°) in the typical graphite oxide structure (sp^2^ hybridization) [37]. This suggested the complete disintegration of the graphite structure to form GO under ultra-sonic vibration. Initially, GO exhibited weight loss of 8.7 wt% at temperature below 150 °C as a result of the loss of absorbed water, while in second stage GO lost more weight (23.6 wt%) in the temperature range of 180–250 °C due to thermal decomposition of oxygen-containing functional groups including hydroxyl and epoxy (Figure S9).

The in vitro toxic effects of GO were determined by measuring cell viability, early and late apoptosis, and necrosis in two well-characterized lung cancer cell lines at different concentrations of GO (5–500 µg/mL). We measured both early and late apoptosis, where the latter can be distinguished from the former by the presence of a disintegrated cell membrane (detected by PI internalization).

**Figure 2:**
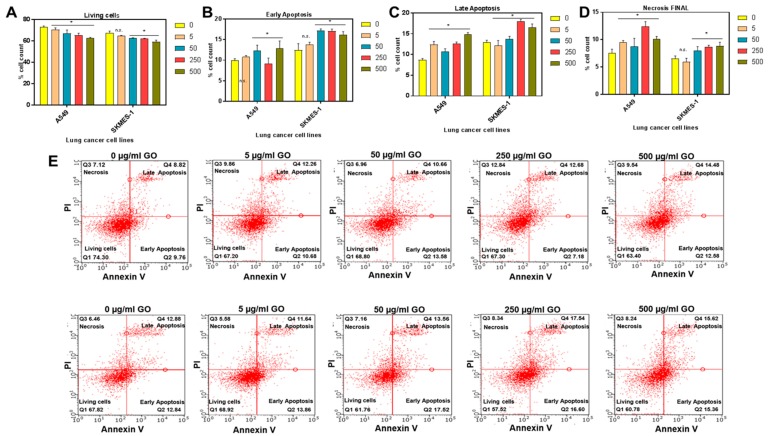
The percentages of living, apoptotic and necrotic lung cancer cells (A549 and SKMES-1) ± graphene oxide (GO) treatment. Cells were stained with annexin V (apoptosis) and propidium iodide (PI; late apoptosis and necrosis) following 24 h of treatment with varying concentrations of GO (0–500 µg/mL) and was assessed by flow cytometry and analysed using Guava 3.1.1 software. Percentage cell counts are shown for (**A**) living cells, (**B**) early apoptosis, (**C**) late apoptosis and (**D**) necrosis at increasing concentrations of GO. Data from three independent experiments are presented as mean ± SD. Groups are indicated as n.s and * *p* < 0.05, representing the outcomes of statistical tests vs control (0 µg/mL). (**E**) Shows scatterplots from one representative experiment in A549 (upper panel) and SKMES-1 (lower panel) cells.

Figure 2A demonstrates a slight but significant (*p* < 0.05) reduction in cell viability of both A549 and SKMES-1 cells after 24 h GO exposure statistically at concentrations of 250 and 500 µg/mL, compared to the control group (0 µg/mL). Significant early apoptosis was also detected (Figure 2B), in A549 cells at 500 µg/mL of GO (*p* < 0.05) compared to controls (0 µg/mL), and in SKMES-1 cells at 50 and 250 µg/mL of GO (*p* < 0.05) compared to controls. Late apoptosis (Figure 2C) and necrosis (Figure 2D) measurements were also carried out for A549 cells. Interestingly, in SKMES-1 cells, 250 and 500 µg/mL of GO significantly induced late apoptosis while necrotic cells were detected at GO concentrations of 50–500 µg/mL. Figure 2E illustrates the representative analysis of one flow cytometry experiment in SKMES-1 and A549. GO induced apoptosis and necrosis at concentrations higher than 50 µg/mL in both cell lines. However, the percentage count of apoptotic cells remained higher compared to necrosis, suggesting that GO may not cause significant damage to the cell membrane, allowing only the binding of annexin V to PS on the cell surface membrane. This indicates that the cell death observed at higher concentrations of GO is probably due to apoptosis rather than necrosis.

For the toxicity exposures undertaken, GO has been shown to be less toxic than other forms of graphene such as reduced GO, which we recently reported for similar cell lines [38]. However, GO has proven to be more toxic than graphene quantum dots as reported by Zhu et al. [39] where it was demonstrated that quantum dots have little toxicity to MG63 (80–90% of cell viability at low dose). This may be because dots are smaller than GO, and hence cause less damage to the cell membrane. GO has been proven to be less cytotoxic, with a reduced free radical production, and cell death compared to reduced GO because of the two-dimensional nature of thin sheets, functional groups and surface charges of GO that allows its efficient cellular uptake [40]. Oxidative stress is thought to be a key factor resulting in graphene toxicity, reducing the number of viable cells and hindering uptake of essential proteins and nutrients [41]. Furthermore, GO may induce various levels of toxicity in in vitro and in vivo models as a result of concentration and dosage patterns, administration routes, entry paths and accumulation of GO via barriers, distribution among different organs, cellular uptake, localisation and clearance [42]. These biological mechanisms depend on physio-chemical properties, sheet size, shape, lateral dimension, functional groups, surface charge and hydrodynamic diameter of the GO. It is evident that a sheet size of GO below 40 nm does not cause off-target toxicity [43,44,45]. We have explored the in vitro toxicity of GO in cancer cells at various concentrations, giving insights into the safe and biocompatible doses of GO to be used for the adsorption and clearance of enzymes. Our results demonstrate that GO at low concentrations did not exhibit obvious toxicity and did not interrupt the course of cell metabolism, gene transcription or cell death. Owing to the flake-like shape of the GO sheet, a readily available surface area is provided to adsorb enzymes while remaining non-toxic to healthy surrounding tissues. However, the neurotoxicity and neuroprotection of GO towards brain cells remain largely unknown and still need further research to explore the possible mechanism of interaction between GO and brain cells, and the capacity of GO not to cross the blood-brain barrier, in improving therapeutic responses to GO.

### 2.2. Basic Characterization of Enzymes

**Figure 3:**
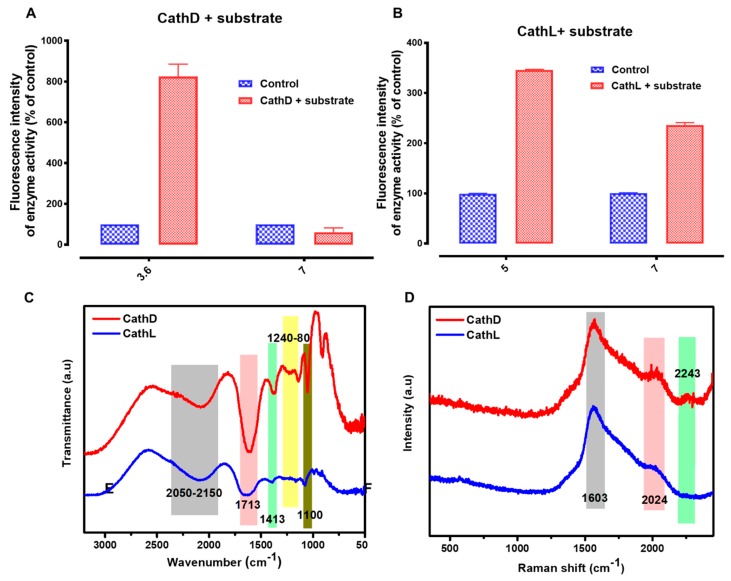

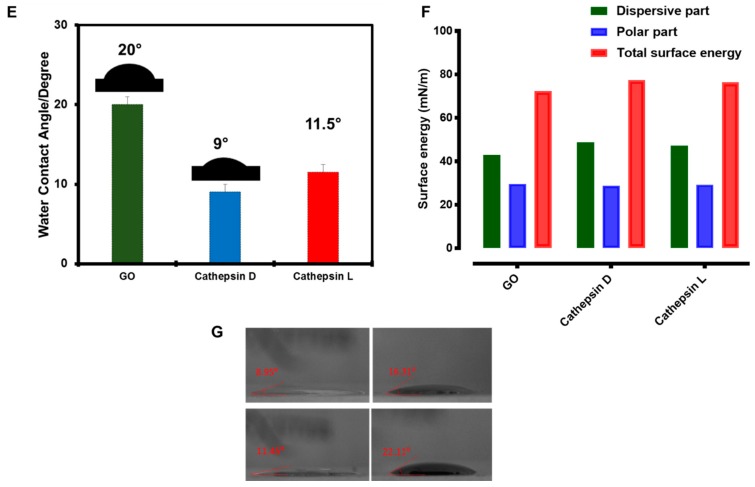
Characterization of cathepsin D (CathD) and cathepsin L (CathL). (**A**) CathD is highly active at pH 3.5–4 (optimum) and inactive at pH 7 and above. The CathD-specific fluorogenic substrate (10 µM) was incubated ± CathD (50 ng/mL) at the pH’s 3.6 and 7, and fluorescence intensity was measured at Ex/Em: 320/393 nm. Each data point represents *n* = 4 experiments. The horizontal bars represent SDs. (**B**) CathL is highly active in ionic buffer. A specific fluorogenic substrate ZVA (5 nM) was incubated ± CathL (50 ng/mL) at pH’s 5 and 7, and its fluorescence signals were measured at Ex/Em: 365/440 nm. Control wells contained substrate alone. The data are presented here as percentage of control (the relevant 100%). Each data point represents *n* = 4 experiments. The horizontal bars represent SDs. (**C**) FTIR spectra of CathD and CathL. (**D**) Raman spectra of CathD and CathL show bands at 1602 and 2024 cm^−1^. (**E**) Water contact angle profile of GO, CathD and CathL gives the values of 20°, 9° and 11.5°. (**F**) Surface energy profile of GO, CathD and CathL. (**G**) Representative image of wettability quantification as measured by water and diiodomethane contact angles of GO, CathD and CathL. The images were taken using a digital camera and analysed for contact angle measurements using the ImageJ processing program.

The proteolytic activity of CathD was investigated using a specific fluorogenic substrate at the two pHs, 3.6 and 7, while the proteolytic activity of CathL was investigated using the CathL-specific fluorogenic substrate ZVA (5 nM) at the two pHs 5 and 7. CathD was more active at pH (3.6). When the pH was increased to 7, the fluorescence signals were observed to be reduced (Figure 3A). The result suggests that CathD is not proteolytically active at neutral pHs. On the other hand, CathL was more active at pH 5.5 (Figure 3B). Fluorescence signals remained almost two times higher than the control at pH 7 (pH of the cell culture medium). The data suggested that CathL is proteolytically active at pH 7.

The representative FTIR spectra of CathD and CathL are given in Figure 3C for the spectral range (3200–500 cm^−1^). In the case of CathD, most of the observed bands appearing at 1100, 1243, 1280, 1413, 1713, and 2050–2150 cm^−1^ are C−O stretch, CH wagging, C−O stretch, carboxylate ion (COO^−^) symmetry, C=O stretch carboxylic acid and C−H alkyl stretch respectively [46,47]. The most prominent band assignments of CathL at 1100, 1243, 1280, 1413, 1713, and 2050–2150 cm^−1^ are C−O stretch, CH wagging, C−O stretch, carboxylate ion (COO^−^) symmetry, C=O stretch carboxylic acid and C−H alkyl stretch respectively [46,47,48]. These bands were not observed in control experiments without CathD/CathL and substrate agents (data not shown). The regions with the widest ranges and their corresponding spectral signatures have been given in Table S1. The representative Raman spectra of CathD and CathL are given in Figure 3D for the spectral range (500–2500 cm^−1^). The most prominent band set of the CathD are 2243, 2024 and 1603 cm^−1^ while assignments of CathL are 2024 and 1603 cm^−1^. The prominent peak at 1608 cm^−1^ relates to the known bands for the Fmoc group as reported earlier [49]. The Raman bands at 2024 and 2243 cm^−1^ could be assigned to the C≡C stretching vibration, which was present in the propargyl group [49]. The surface free energy and its polar and dispersive parts were calculated to investigate the binding capacity and weight of electrostatic and/or van der Waals interactions between GO and the enzymes. The binding capacity of GO, CathD and CathL were calculated using the contact angle method and their respective contact angles have been shown in Figure 3E. The surface free energies, polar and dispersive parts of GO, CathD and CathL are shown in Figure 3F. CathD has the highest total surface energy of 77.4 mN/m, although GO, CathD and CathL have similar trends of surface energies of total and their respective parts because of the similar amount and weight of functional groups. As a result, the use of GO as an adsorbent could allow enzymes to be adsorbed and substituted to improve the binding of CathD/CathL with GO. Therefore, it appeared that amino-acid replacement at the basal planes of GO can lead to protein-ligand binding. (See set of “snapshots” in Figure 3G).

### 2.3. Enzyme Interaction with GO

**Figure 4:**
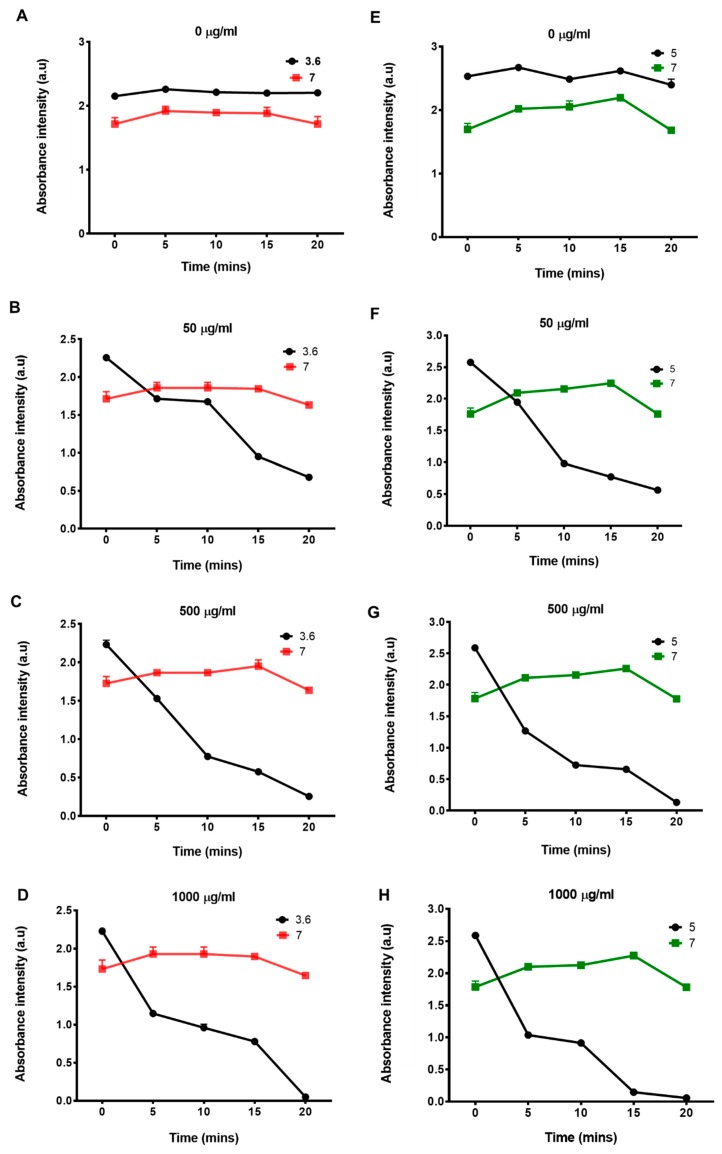
Effect of various concentrations of GO on CathD and CathL activities. Absorption of CathD (**A**–**D**) and CathL (**E**–**H**) by GO at different concentrations (50, 500, and 1000 µg/mL) incubated for 2, 5, 10, 15, and 20 min. Absorbance signals were measured using a PHERAstar BMG plate reader at λ = 280 nm. Each data point represents *n* = 4 experiments. The horizontal bars represent SDs. For some data points, the error bar is smaller than the diameter of the data point.

**Figure 5:**
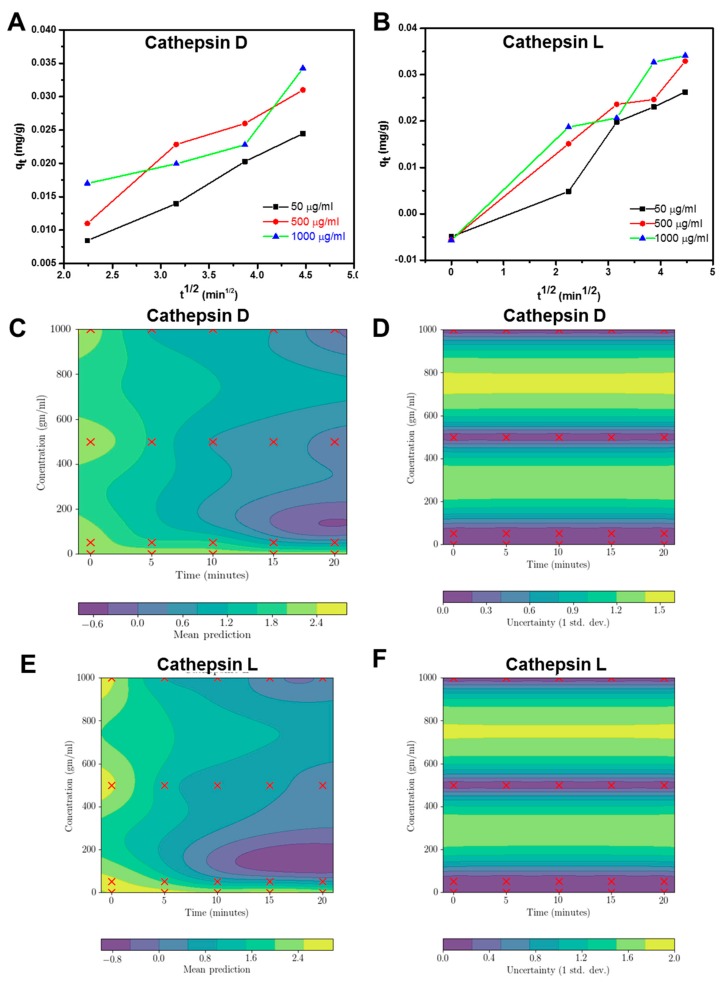
(**A**,**B**) Kinetic models fitting to the data for CathD and CathL using piecewise linear regression analysis of experiments in which (**A**) CathD and (**B**) CathL were adsorbed onto GO. (**C**–**F**) Gaussian process regression models to find the prediction and uncertainty in adsorption of CathD (**C**,**D**) and CathL (**E**,**F**) relating independent variables (time and concentration) to the dependent variable (absorption). In (**C**) and (**E**), the mean predictions are depicted, and the uncertainty in predictions is shown in (**D**) and (**F**).

**Figure 6:**
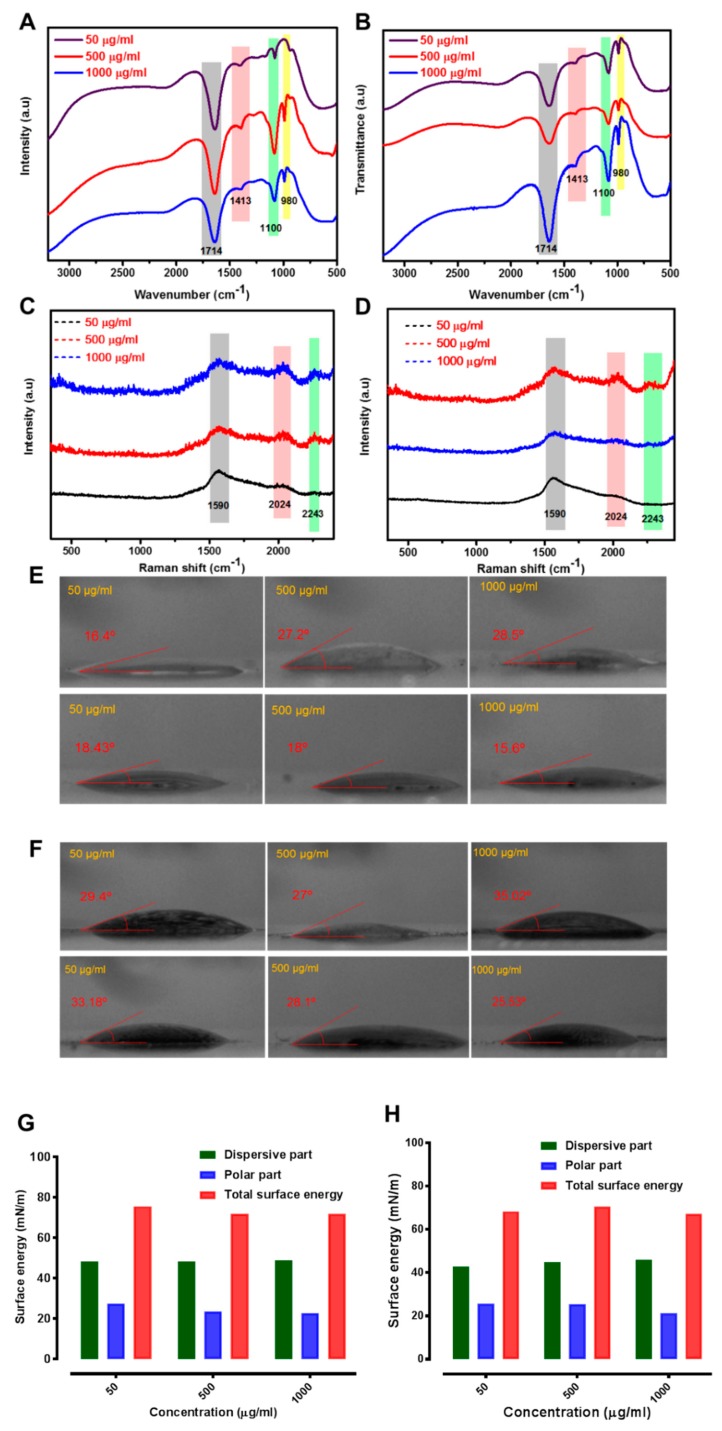
(**A**,**B**) FTIR spectra of CathD/CathL-linked graphene oxide (GO) at 50, 500 and 1000 µg/mL concentrations of GO. (**C**,**D**) Raman spectra of CathD/CathL-linked GO at 50, 500 and 1000 µg/mL GO concentrations. (**E**,**F**) Contact angle profiles of CathD/CathL-linked GO interfaces at 50, 500 and 1000 µg/mL concentrations of GO. The diiodomethane contact angle was determined to calculate the surface energy of the enzymes, GO and the interfaces of GO with enzymes. (**G**,**H**) The surface energy profile of GO-CathD/CathL interfaces which have three segments of total surface energy, dispersive surface energy and polar surface energy—the profiles correspond to 50, 500 and 1000 µg/mL concentrations of GO which had been treated with CathD and CathL.

Batch adsorption studies were performed to measure the effect of pH on the adsorption process of CathD and CathL using GO as an adsorbent. Figure 4 shows absorbance variations at different concentrations of GO (0, 50, 500 and 1000 µg/mL) mixed with CathD and CathL at different time points (0–20 min). The decrease in absorbance signals of CathD and CathL at pH’s of 3.6 and 5, respectively revealed the amount of CathD and CathL adsorbed to GO. At optimal incubation times and concentrations of GO, CathD and CathL were almost fully adsorbed onto the GO surface. The CathD and CathL adsorption process was found to be pH-dependent and concentration-dependent, demonstrating that the highest adsorbed amounts were at more acidic pHs (3.6 and 5). For pH 3.6, the amount of CathD adsorbed increased from 50 to 1000 µg/mL over a time scale of 0 to 20 min. The adsorption capacity of GO (1000 µg/mL) was above 90% after 20 min. CathL adsorption onto the GO surface followed a similar pattern at pH 5 and at 1000 µg/mL GO the highest value of efficiency was attained after 20 min. Figure 5 shows that an increase in adsorption capacity occurred for both enzymes over a 20 min time period, reaching a maximum capacity of above 90%. The capacity was found to be slightly greater for higher concentrations of GO. The results are in good agreement with experimental data (Figure 6A,B). 

Adsorption kinetics are useful to evaluate the adsorption process and adsorption rate. In this study, we have used an intraparticle diffusion model. This multi-linearity graph plot of intraparticle diffusion shows three segments: the first portion is the instantaneous adsorption segment which shows the adsorption of external surface of GO; the second step is the gradual adsorption stage assigned to intraparticle diffusion; and the third straight portion depicts the final equilibrium stage due to the low adsorbate concentration left in the solution. Figure 6A,B show that the intraparticle diffusion plot at each concentration did not pass through the origin, indicating that the intraparticle diffusion was not the only rate-controlling process. This is indicative of an additional role of boundary layer diffusion control. The intraparticle diffusion constant values are shown in Table S2. Gaussian process regression models for CathD and CathL relating independent variables (time and concentrations) to the dependent variable of adsorption are shown in Figure 5C–F. In Figure 5C,E, the mean predictions for CathD and CathL are depicted respectively, and the uncertainty in these predictions has been shown in Figure 5D,F. The mean predictions for CathD indicate that promising (lower) absorption can be achieved with a concentration of around 100 µg/mL when CathD is incubated with GO for 15 to 20 min. The models also revealed that concentrations of CathD greater than 900 µg/mL which are incubated for about 18 min could also be promising. Figure 5E,F show similar trends for CathL. The fluorescence of GO only has been shown in Figure S10. Figure S11 illustrates the measurement of normalised fluorescence intensities of various concentrations of GO (0, 50, 500 and 1000 µg/mL) exposed to CathD and CathL at different time points (0–20 min). CathD and CathL were enzymatically cleaving a GO substrate which gave rise to a fluorescent product, at increasing concentrations of GO (0, 50, 500 and 1000 µg/mL). GO was able to concentration-dependently increase the catalytic activity of CathD and CathL at all pHs tested. A slight difference in emission spectra also occurs, suggesting that lower pH values represent an improved exposure of non-polar sites of adsorbate in the enzymes. Fluorescence loss was observed in the case of CathL at pH 7, due to the reversible nature of CathL inhibition. The lack of any significant difference in fluorescence signals might be because of the adsorption of enzymes induced by GO. This uptake allows localization of internalized GO under different pH conditions [50,51]. This uptake could be attributable to the large size of GO which blocks fluorescence signals. The binding of CathD and CathL to GO shows that both the enzymes and GO sheets form a new tertiary structure. These tertiary structures may contribute significantly to the self-fluorescence characteristics of GO. Understanding the pre-clinical and clinical effects of such factors to allow the development and adsorption of pro-tumorigenic and pro-metastatic enzymes, released into the extracellular matrix from malignant tumors, will be the focus of further studies.

FTIR and Raman spectroscopic findings can be used to monitor the macromolecular movements and vibrational/rotational states of specific chemical groupings which bind target biomolecules with high specificity during the formation of the nano-bio-interface of CathD- and CathL-GO. Figure 6A,B illustrates FTIR spectra of GO-CathD/CathL at the concentrations of 50, 500 and 1000 µg/mL of GO after 20 min. The FTIR spectrum of CathD-linked GO revealed a range of CathD and CathL absorption bands including C=O (υ_C=O_ at 1714 cm^−1^), and the peak of the C−N stretch mode (υ_C=O_ at 1100 cm^−1^) at all the concentrations represents the CH stretching and NH bending. The specific band of GO-CathD (at two concentrations of 500 and 1000 µg/mL GO) (Figure 6A) showed the characteristic peak of an alkoxy group at 980 cm^−1^ which is associated with the C=O functional groups of GO and CathD. The peaks at 1413 cm^−1^, ascribed to NH bending and CN stretching, also confirmed the presence of CathD and CathL. Figure 6C,D show Raman spectra of GO-CathD/CathL. The amide-1 vibration at 1590 cm^−1^ arose from the typical υ_C=O_ stretching vibration. The band in the range of 2020–2250 cm^−1^ was assigned to the specific C−H_3_ and C−H_2_ deformation vibrations which mainly arose from the side chains of different amino acids. The band in the range of 1200–1340 cm^−1^ was assigned to the amide-III vibration which typically arose from the combination of the N-H bending and C−C stretching vibration [52,53]. In the Raman spectra of GO and GO-CathD/CathL, the slight shifting of peaks towards lower wavenumber can be observed. GO has two typical peaks at ca. 1355 cm^−1^ and 1580 cm^−1^. The spectra at 1600–1620 cm^−1^ can be assigned to the C=O stretching of carboxylate and C−H_2_ deformation vibrations. Based on these bands, it is concluded that the CathD and CathL interacted with GO through its amide groups. Both cathepsins have a deep bonding pocket with the binding groups identified by FTIR, which are held together with GO by electrostatic attractions.

The functional groups present at the outermost surface of GO readily facilitate its coverage with inert molecules, which increases surface hydrophilicity and subsequently enhances the bonding strength of these nanostructures [54]. Several site-specific variants of GO have been employed in attempts to alter the surface-inactivation of ‘wild-type’ enzymes. The extent of enzyme absorption (and the mechanistic insight this provides in relation to the proteins’ interactions with surfaces) have been probed by water contact angle (WCA) measurements and surface energy determinations (Figure 6E,F). The CathD and CathL displayed higher binding activity towards GO, as demonstrated by the WCA values. Upon CathD and CathL interactions, the WCA profiles of GO shifted to higher values, suggesting that a good level of surface hydrophilicity was achieved (Figure 6F). The effect was more pronounced for the higher concentrations, whose average WCA value increased by 8.5 and 15.0 degrees for CathD and CathL, respectively. The changes in diiodomethane contact angle (Figure 6F) showed the surface energy profile (Figure 6G,H). The binding free energies of GO to CathD and CathL are shown in Figure 6G,H. The intermolecular vdW and electrostatic interactions are believed to improve the desolvation process because of the substitution of one oxygen-containing functional group with an amino group, which in turn increases the total free energy of the compound. However, the polar penalties upon binding of these two proteins to GO were decreased.

These findings have provided significant information about the surface interactions of GO sheets with CathD and CathL: (i) the number and amount of the functional groups and their reactivity; (ii) the critical role of surface hydrophobicity in the adsorption process. The WCA of CathD and CathL is shown in Figure 3. Moreover, the key differences in the amount and number of functional groups and bonding affinities are responsible for the rise in total and dispersive surface energy. The low polar and high dispersion parts (Figure 6G,H) of the surface energy profiles revealed that the polar and nonpolar side-chains of CathD/CathL facilitate conformational alterations in the CathD/CathL structure, which in turn lead to a high adsorption capacity of GO for CathD/CathL.

## 3. Discussion

Potential cancer therapies include the development of innovative treatment modalities that are capable of clearing the pro-tumorigenic enzyme by developing a novel platform based on biocompatible adsorbents. The currently available mainstream treatment options have resulted in improved survival and quality of life, although ovarian and breast cancers remain progressive diseases. Thus, there is an ever-growing need for the development of alternative approaches. Conventional biological drug therapies have limitations due to unwanted side effects on normal tissues/cells that adversely affect the efficacy and safety of the treatment. The emerging paradigm of personalised and precision medicine provokes the concept that adsorption of these enzymes in the local tumor environment could be achieved by using porous adsorbents. Enzyme-targeted therapy provides a great opportunity for this by addressing the mechanisms of pro-tumorigenic enzyme clearance and therapeutic action. In this study, we developed a GO that breaks down and takes up enzymes which promote increased invasiveness and metastasis. The surface charge, surface area, chemical reactivity and electronic characteristics of GO were used to target these enzymes with sustained release of functional groups, free radical and porous sites for entrapment of CathD and CathL. The inhibition of CathD and CathL was observed at specific pH values which support metastasis. Inhibition of CathD and CathL was verified by enzyme activity using specific substrates. The analysis of the released CathD and CathL libraries was carried out using a wide variety of analytical tools such as FTIR, Raman, WCA and surface energy profiles (see Figure 2; Figure 6), thus posing a significantly fast-tracked identification procedure and considerable output compared with conventional tools to analyse nanoparticle interactions with proteins. In this manner, the characterization of the studied enzymes for their binding and bioactivity is carried out to better understand their structural and functional behaviours.

The current approach of enzyme targeting offers a number of important advantages over conventional approaches. First, it permits the simplistic tagging of cathepsins with very high transformation/removal efficiencies using GO, which considerably increases the likelihood of recognising biomolecular fractions. Secondly, the clinical relevance and biosafety of this modality would further benefit from utilising a GO system that is already used in clinical trials to introduce drug/gene carrier vehicles. Finally, the approach presented here is greatly adaptable and can be used largely for the innovation of theranostic saviours of disease-associated enzymes. The present work was mainly applied to targeting two ovarian and breast cancer-associated enzymes. The two-dimensional and adsorbing nature of GO could reduce the likelihood of abundance of these enzymes to induce tumor cell invasion and metastasis, and thereby maximize the broad applicability of GO. Furthermore, the GO not only allows for robust interactions with enzymes but also enables the compact packaging of the GO within dissolvable capsules, facilitating non-invasive oral administration to track these proteins, which could be used as a diagnostic tool. Given the clear benefits achievable by using enzyme-targeted therapy (compared with the currently available modalities such as chemotherapy, radiation therapy etc.), the cost-effectiveness involved in producing GO is another advantage for implementing this material as a standard-of-care in the treatment of cancer. Currently, clinical-scale manufacturing of GO entails a range of protocols to fabricate, modify, functionalise, deliver and selectively accumulate and administrate into the living systems. Future work will address cell-based and pre-clinical metastatic disease models and will possibly include further developments to integrate targeted and safe delivery of GO to the tumor sites with sufficient selectivity to facilitate the removal of disseminated enzymes.

## 4. Experimental Section

### 4.1. Synthesis and Characterization

Exfoliated graphene oxide (GO) flakes were synthesized from exfoliated graphite using the modified Hummer’s method as previously reported by us [27,28,38]. NaNO_3_ (1.5 g) and H_2_SO_4_ (150 mL, 98%) were added to a 800 mL round-bottom flask with graphite flakes (2 g). The reaction mixture was mixed under magnetic stirring following by the immersion of the flask in an oil bath. The mixture was then heated at the temperature of 35 °C, before adding KMnO_4_ (9 g) into the flask. The mixture was subjected to constant continuous stirring for 24 h, followed by addition of more H_2_SO_4_ (280 mL, 5%) and the temperature was increased to 85–95 °C. The mixture was stirred for another 2 h before removing the bath. The flask was allowed to cool down to 60 °C. Finally, H_2_O_2_ (15 mL, 30 wt%) was added and the mixture was stirred for another 2 h. The resultant product was washed 7–8 times with HCl (3 wt%) and then washed 4–5 times with distilled water to eliminate any contaminants. As obtained GO was dispersed in water under stirring. As prepared GO was then used for further characterization. Transmission electron microscopy (TEM) (JEOL-2100 TEM, JEOL, Madrid, Spain), at an accelerating voltage of 200 kV) was used to obtain high resolution microstructural images. A drop of the as prepared GO was deposited on a holey carbon Cu grid to prepare the TEM samples. X-ray diffraction (XRD) analysis was conducted using Cu Kα radiation. X-ray measurements were performed at a voltage of 40 kV and a current of 40 mA and spectrum was collected a step size of 0.02° (2θ) and a step time of 1 s. Fourier-transform infrared (FTIR) spectroscopy was conducted by means of a Tensor-27 FTIR spectrometer (Bruker Optics, Champs-sur-Marne, France) in the wavenumber range of 4000–500 cm^−1^. FTIR samples were prepared by mixing the sample with KBr. Raman spectroscopy was carried out with laser excitation at 532 nm (Renishaw, Stroud, UK). To calculate the surface charge of GO, zeta potential measurements were performed using a colloidal dynamics zeta probe. UV/Vis spectrophotometry was performed using a 6715 UV–Vis instrument (Jenway, Staffordshire, UK).

### 4.2. Cell Viability

Cell viability experiments using flow cytometry has been described elsewhere [34]. Briefly, cells were treated with or without 5, 50, 250, 500 and 1000 µg/mL of GO for 24 h. After trypsinisation, cells were stained with annexin V (BioLegend, London, UK) and propidium iodide (PI, Sigma-Aldrich, Gillingham, Dorset, UK)) and subjected to flow analysis using a Guava flow cytometer (Millipore UK Limited, Hertfordshire, UK). The data were analysed using the Guava 3.1.1 software. The experiment was carried out at least three times and the data obtained were analysed using GraphPad Prism 5.04 (GraphPad Software, San Diego, CA, USA), and expressed as % cell count ± SD, Mann Whitney. 

### 4.3. Regression Model

We were particularly interested in determining a good estimate of the most effective level of concentration of CathD and CathL and the experiment time required for biological applications. As such we built a model that may indicate which pair of concentrations and times are promising and subsequently help in our decision making. However, we had limited data (due to the expense of conducting many experiments) and repeated measurements are always noisy (in that we obtained a slightly different measurement for the same concentration and time). Therefore, it is of paramount importance to consider these uncertainties in modelling the performance of these enzymes. Standard non-linear regression models usually predict the general trend without capturing such uncertainties. As an alternative, we used Gaussian processes (GPs) to model absorption (dependent variable) with respect to time and concentration (independent variables) for both CathD and CathL. A GP model allowed us to inspect the expected performance and the predictive uncertainty for the enzymes. Thus we were able to strike a balance between predicted performance and uncertainty to make an informed decision.

Formally, a GP may be considered as a collection of random variables, which is jointly Guassian distributed [38,52]. This essentially allows us to encapsulate the intuition that for a small change in concentration and time there should be a small change in performance. Let *D =* {x*_i_*, *y_i_*} be a data set consisting of *n* data points, where the *i-*th vector x*_i_* consists of a time and a concentration (independent variables), and *y_i_* is the associated absorption (dependent variable). A trained GP model then produces the following posterior predictive Gaussian distribution: *P*(*y_n_*_+1_|x*_n_*_+1_, *D, θ*) ~ *N*(*μ*(x*_n_*_+1_), *σ*(x*_n_*_+1_)), where *θ* is a set of hyperparameters that are optimised using collected data *D*, *μ*(x*_n_*_+1_) is the expected performance for x*_n_*_+1_, and *σ*(x*_n_*_+1_) is the predictive uncertainty. The details of training a GP model can be found in [52].

### 4.4. Water Contact Angle Measurements and Surface Energy Calculations

A contact angle goniometer was used to calculate the wettability of GO, CathD and CathL. A digital camera was used to capture the images and the contact angle was measured using the ImageJ processing program. The contact angle surfaces were developed by dropping a 10 µL drop onto a glass slide. The surface free energies were estimated by quantifying the contact angle of diiodomethane (DIIO) on the surface of the sample. 10 µl drop of DIIO was used in each measurement. The surface free energy of a solid sample is expressed by Young’s equation, where S is solid and L is liquid:

*σ*_S_ = *σ*_SL_ + *σ*_L_ × cos*θ* (1)

where *σ*_L_, and *σ*_SL_ represent the surface tension of the liquid and the interfacial tension between the liquid and the solid, respectively and *θ* is the contact angle shaped by the liquid on the surface of the sample respectively. Here, we are measuring *σ*_S_ with the help of known value of *σ*_L_ and unknown value of *σ*_SL_. According to the Fowkes method [53], the surface tension is given by:

*σ*_SL_ = *σ*_L_ + *σ*_S_ – 2((*σ*_L_^D^ × *σ*_S_^D^)^1/2^ + (*σ*_L_^P^ × *σ*_S_^P^)^1/2^) (2)

where the surface free energies are mixture of dispersive (D) and polar (P) parts together. This would be used to exclude the unknown value in Equation (1).

The polar part of liquid is zero for DIIO, so:

*σ*_S_^D^ = *σ*_L_ × (cos*θ* + 1)^2^/4 (3)

where *σ*_L_ = *σ*_L_^D^ = 50.8 mN/m. The dispersive part of the surface free energy of the sample can directly be found from the contact angle.

The polar and dispersive parts of water are: *σ*_L_^D^ = 26.4 mN/m and *σ*_L_^P^ = 46.4 mN/m. Equations (1) and (2) can be reorganised to calculate the polar part of the surface energy of the sample:

*σ*_S_^P^ = (*σ*_L_ × (cos*θ* + 1)/2 – (*σ*_L_^D^ × *σ*_S_^D^)^1/2^)^2^/*σ*_L_^P^ (4)

If the values of the dispersive and polar parts are known, the total surface energy of the sample will be:

*σ*_S_ = *σ*_S_^D^ + *σ*_S_^P^ (5)

Contact angles of water and DIIO on surface of GO are 33.4° and 20° respectively. The dispersive component, polar component and total surface energies of GO are 42.8, 29.6 and 72.4 mN/m respectively [53]. The other surface energies were calculated in the same manner.

### 4.5. Proteolytic Activities

Citrate and phosphate buffer solutions were prepared at pHs 3.6, 5.0 and 7.0. Their composition is given in Supplementary notes 2 and 3. The measurement of proteolytic activities has been described elsewhere [18,20]. A brief description of each enzyme activity is presented below:

### 4.6. CathD Experiment

The buffers required to test CathD-proteolytic activities contained 0.005% of Tween20 (Sigma-Aldrich) and the pHs were adjusted to 3.6 and 7. CathD-fluorogenic substrate (100 nM; Enzo Life Sciences, Exeter, UK) was incubated ± CathD (50 ng/mL; recombinant from human liver, Sigma-Aldrich) and plates were read after 60 s of shaking at Ex/Em: 320/393 nm.

### 4.7. CathL Experiment

The buffers contained 1 mM DTT to disrupt the disulfide bonds, resulting in an active enzyme. CathL fluorogenic substrate Z-Val-Val-Arg-AMC (ZVA; 5 nM) was incubated ± CathL (50 ng/mL; recombinant from human liver, Sigma-Aldrich) at pHs 5 and 7. The plate was shaken for 60 s in a plate-reader prior to fluorescence reading at Ex/Em: 365/440 nm.

Both enzyme activities were measured using a SpectraMax plate reader (Molecular Devices UK Limited, Berkshire, UK). The data was normalised to control and represented as a percentage of the control.

### 4.8. Enzyme Interaction with GO

An interaction between CathD or CathL (50 ng/mL) and GO (50, 500 and 1000 µg/mL) was tested in pH buffers (pHs 3.6 and 7 for CathD, and pHs 5.5 and 7 for CathL). pH values of 3.6 and 5.5 are optimum for CathD and CathL activity, respectively. CathD and/or CathL was incubated with GO at different concentrations for 2, 5, 10, 15 and 20 min. The experiment was performed in ×4 96 well black opaque plates (Greiner Bio-One Ltd., Gloucestershire, UK). Plates were read at the same time scale as previously mentioned, of incubation at room temperature to measure absorbance at 280 nm for CathD and CathL using a SpectraMAX plate reader. The data normalised to the control and represented as a percentage of this control. The fluorescence intensity of the GO hydrolysis was identified kinetically using a SpectraMax plate reader. This was repeated (*n* = 4) with CathL at different concentrations of GO (50, 500 and 1000 µg/mL). The control wells contained GO only. FTIR, Raman spectroscopy, wettabilities and surface energies were carried out in the same manner as explained in the previous section.

To verify the adsorption of CathD and CathL, an intra-particle diffusion model was used. Fick’s second law was used to find out the intraparticle diffusion model as a rate-determining step during the adsorption experiment. It was carried out to verify whether it may control the kinetics of adsorption process [54,55]:

(6) $$q_{t}=k_{id}\sqrt{t}+I$$

where *I* represents the boundary layer effect (a large value corresponds to a larger boundary layer thickness and *k*_id_ is the intraparticle rate constant and *k_int_* (g/mg min^1/2^) and *C* represent the adsorption constant and the intercept, respectively [56,57]. The intercept is measured from the plot of *q_t_* versus *t^1/2^*.

### 4.9. Statistical Analysis

Statistical analysis was carried out between two groups by the Mann-Whitney test, and between multiple groups were compared by one-way analysis of variance (ANOVA) with Tukey post-hoc testing or two-way ANOVA with a Bonferroni post-hoc test, using GraphPad Prism 5 software. The results are shown as mean ± s.d, (standard deviation) unless otherwise indicated. The value of *p* < 0.05 was considered statistically significant.

## 5. Conclusions

In summary, our findings represent a straightforward and highly reliable approach for the rapid and facile removal of pro-metastasis enzymes. GO with its variable zeta potential, variety of functional groups and very large (and in principle fully accessible) surface area, is an extremely promising candidate for the adsorption of such enzymes. Our results show that this material is compatible with cells. In addition, the adsorbent preparation is based on abundantly available and cost-effective graphite as the main precursor. Graphene oxide nanostructures are straightforward to prepare and are highly stable, which streamlines long-term storage at room temperature and correspondingly eases the manufacturing cost. Therefore, if employed in clinical settings as an innovative platform, this highly adaptable strategy could provide a real-world, cost-effective and broadly relevant procedure to treat chronic and complex diseases.

## Supplementary Materials

The following are available online at https://www.mdpi.com/2072-6694/11/3/319/s1, Figure S1: Transmission electron microscopy image of graphene oxide, Figure S2: Basic characterization of exfoliated graphene oxide (GO). (A) XPS survey. (B) The C_1s_ spectrum of the GO shows three main components arising from C−O (hydroxyl and epoxy, 286.7 eV), C=C/C−C (284.7 eV) and O=C−O (carboxyl, 288.8 eV) and a minor component of the C=O (carbonyl, 287.4 eV) and O=C−OH (289.1 eV) species, Figure S3: Raman spectrum of the graphene oxide sample shows intense D (1358 cm^−1^) and G peaks (1595 cm^−1^) of defects and the in-plane stretching motion of pairs of sp^2^ atoms, respectively, Figure S4: BET surface area of graphene oxide measured by nitrogen sorption isotherms measured at –196 °C. The BET surface area value obtained for this sample using the BET method was 25 m^2^/g, Figure S5: Representative zeta potential of graphene oxide over a range of different pH values, Figure S6: The Fourier transformed infrared (FTIR) spectrum of graphene oxide shows vibrations of functional groups of C−O−C (~1000 cm^−1^), C−O (1230 cm^−1^), C=C (~1620 cm^−1^), C=O (1740–1720 cm^−1^) bonds and O−H (3600–3300 cm^−1^), Figure S7: (A) UV/Vis absorption spectra of graphene oxide solutions with different concentrations (from 0.039–10 mg/mL) show the main peak around 232 nm. (B) The plot of the absorbance (λ = 232 nm) divided by the cell length, versus the concentration. The Lambert–Beer law (A = α × C × l), allowed the determination of the absorption coefficient (α). This linear relationship fits well with the Lambert-Beer Law, indicating the good water solubility of the GO product, Figure S8: The XRD pattern recorded from graphene oxide shows a (001) peak at 2*θ* of 13.7°, Figure S9: TGA of exfoliated graphene oxide. TGA was performed in the nitrogen atmosphere, Figure S10: Photoluminescence emission spectrum of graphene oxide, Figure S11: Effect of different concentrations of GO on CathD and CathL fluorescence activities. GO at different concentrations (50, 500, and 1000 µg/mL) were incubated with CathD (A, B) and CathL (C, D) in 96 well plates at different time-points (2, 5, 10, 15, and 20 min) as shown where RFU is relative fluorescence units. Fluorescence signals were determined using a plate reader at Ex/Em: 355/450 nm. Each data point represents the mean of *n* = 4 experiments. Bars show SDs, Table S1: Characteristic IR bands of the protein linkages, Table S2: Kinetic parameters obtained for CathD and CathL for GO using an intraparticle diffusion model.
