# Supplementary material for: Graphene Oxide-Based Targeting of Extracellular Cathepsin D and Cathepsin L As A Novel Anti-Metastatic Enzyme Cancer Therapy
Source: Cancers (Basel). 2019 Mar 6;11(3):319. doi: 10.3390/cancers11030319 (PMC6468385; doi:10.3390/cancers11030319)
Supplement: Supplementary file 1 [file cancers-11-00319-s001.pdf]

cancers

MDPI

Supplementary Materials: Graphene Oxide-Based Targeting of Extracellular Cathepsin D and Cathepsin L As A Novel Anti-Metastatic Enzyme Cancer Therapy

Tanveer A. Tabish, Md Zahidul I. Pranjol, David W. Horsell, Alma A. M. Rahat, Jacqueline L. Whatmore, Paul G. Winyard and Shaowei Zhang

Chemicals and reagents

$NaNO_3$ (product no. S5506), H_2SO_4 (95.0–98.0%, product no. 320501), $KMnO_4$ (product no. 223468), H_2O_2 (30 wt%, product no. 216763), graphite flakes (product no.17346-25) and HCl (36 wt%, product no.: 7647-01-0) used in this work were purchased from Thermo Scientific, Fisher Scientific, Acros, Nacalai Tesque and Alfa Aesar, respectively.

Supplementary Note 1:

We first characterized graphene oxide (GO) with a wide range of characterization tools such as Fourier-transform infrared spectroscopy (FTIR), Raman spectroscopy, transmission electron microscopy (TEM), scanning electron microscopy (SEM), Zeta potential analyser, thermogravimetric *analysis* (TGA), X-ray diffraction (XRD) and Brunauer−Emmett−Teller (BET) surface area method.

The surface area of the GO as measured by the N_2 absorption Brunauer−Emmett−Teller (BET) method is 25 m^2/g having a pore volume of 0.07 cm^3/g (Figure S4). However, it is still lower than the theoretical specific surface area for completely exfoliated and isolated graphene sheets (~2620 m^2/g), potentially because it measures the outer surface of GO grains. The nitrogen molecules are inaccessible to the interlayer and interlamellar spaces of GO and as a result acid-base processes in aqueous GO dispersions take place on much greater surfaces. According to the Ruess model, graphite oxide consists of wrinkled carbon sheets composed of trans-linked cyclohexane, and the fourth valencies of the carbon atoms are bound to axial OH-groups and ether oxygen atoms in 1,3-positions. As a result, this geometrical network, functional groups existing at the edges and basal planes of GO sheets, degree of exfoliation and dispersion, and surface chemistry of GO hinders nitrogen access/adsorption to inner surfaces of GO, which is generally opened up upon exfoliation. The hydrophilic oxygen-containing functional groups provide GO sheets with a good dispersibility in water. The GO obtained shows good water solubility (Figure S6) and exhibits ultraviolet-visible (UV/Vis) absorption spectra of the GO at the absorption peak at 232 nm, which is attributable to a π–π^* transition of the C=C bonds.

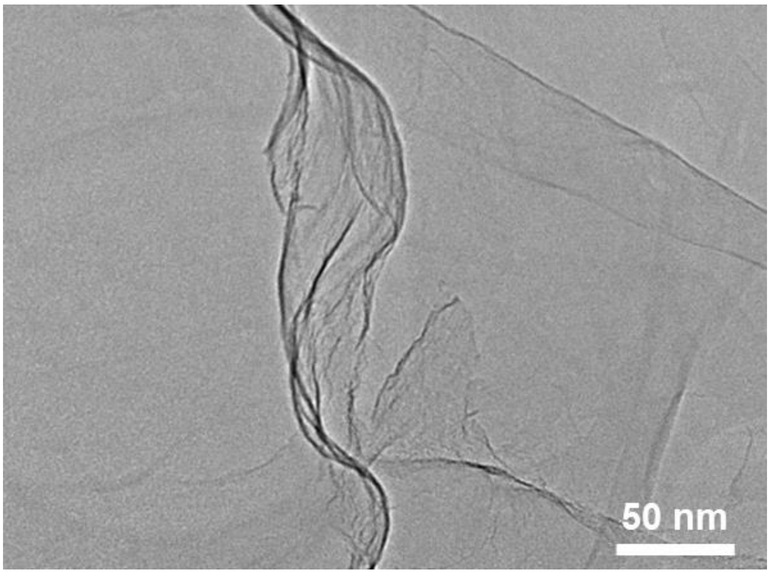

Figure S1. Transmission electron microscopy image of graphene oxide.

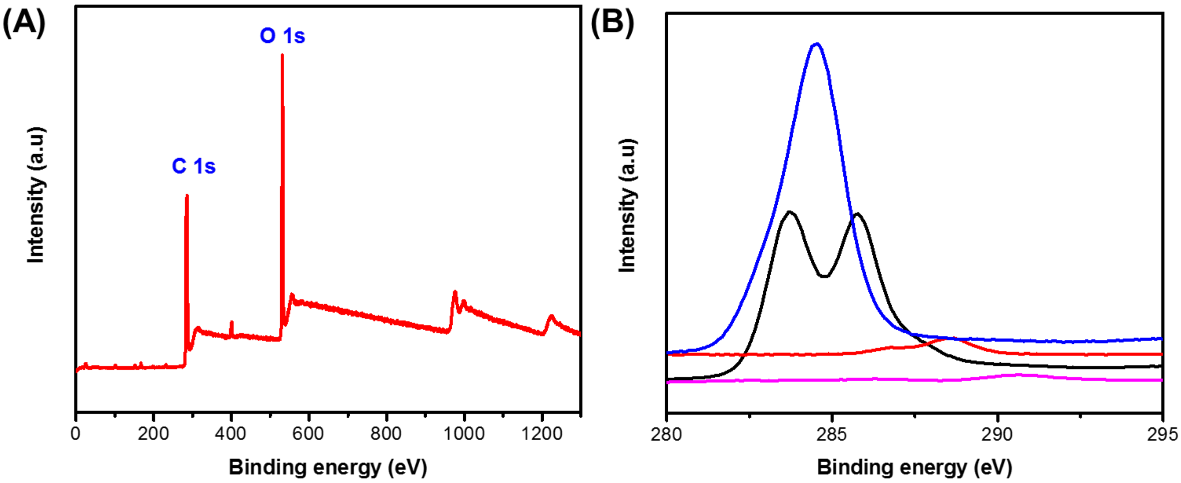

Figure S2. Basic characterization of exfoliated graphene oxide (GO). (**A**) XPS survey. (**B**) The C_{1s} spectrum of the GO shows three main components arising from C–O (hydroxyl and epoxy, 286.7 eV), C=C/C–C (284.7 eV) and O=C–O (carboxyl, 288.8 eV) and a minor component of the C=O (carbonyl, 287.4 eV) and O=C–OH (289.1 eV) species

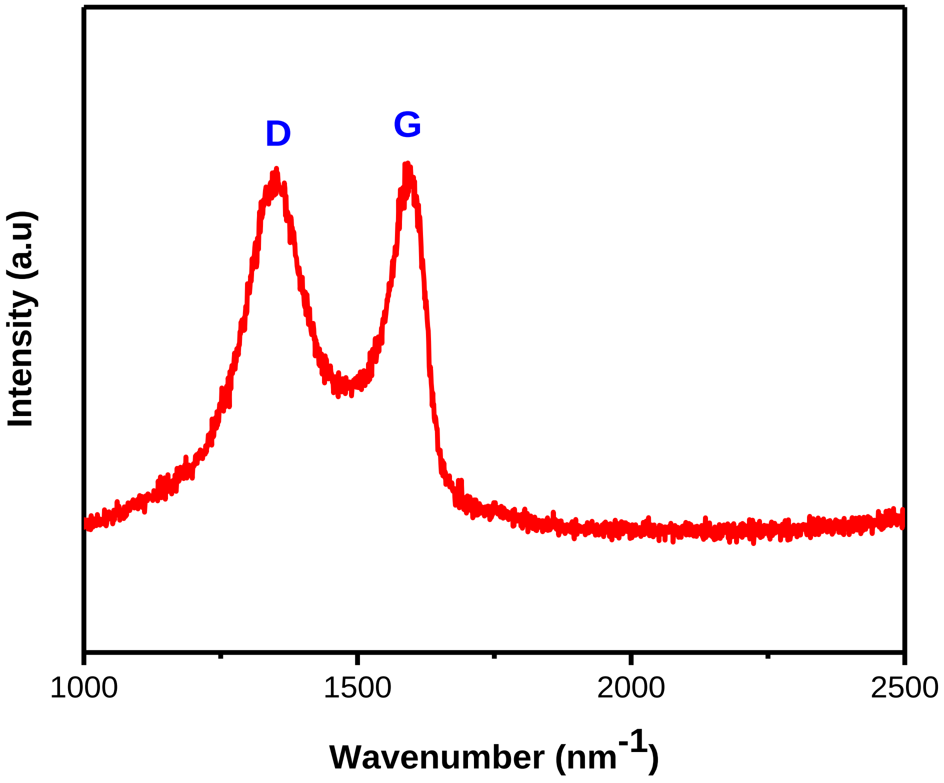

Figure S3. Raman spectrum of the graphene oxide sample shows intense D (1358 cm^{-1}) and G peaks (1595 cm^{-1}) of defects and the in-plane stretching motion of pairs of sp^2 atoms, respectively.

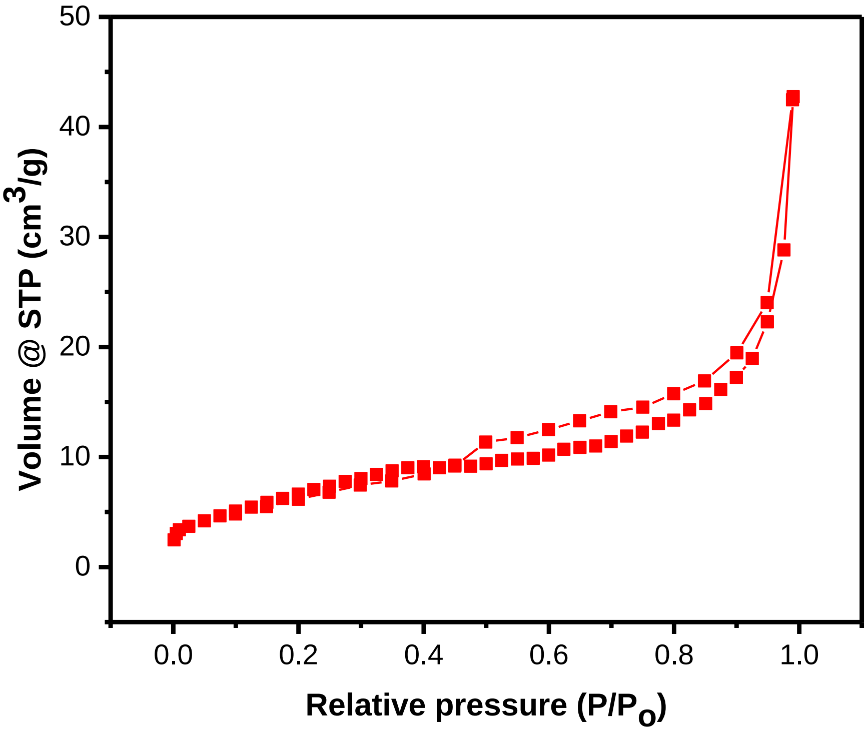

Figure S4. BET surface area of graphene oxide measured by nitrogen sorption isotherms measured at −196 °C. The BET surface area value obtained for this sample using the BET method was 25 m^2/g.

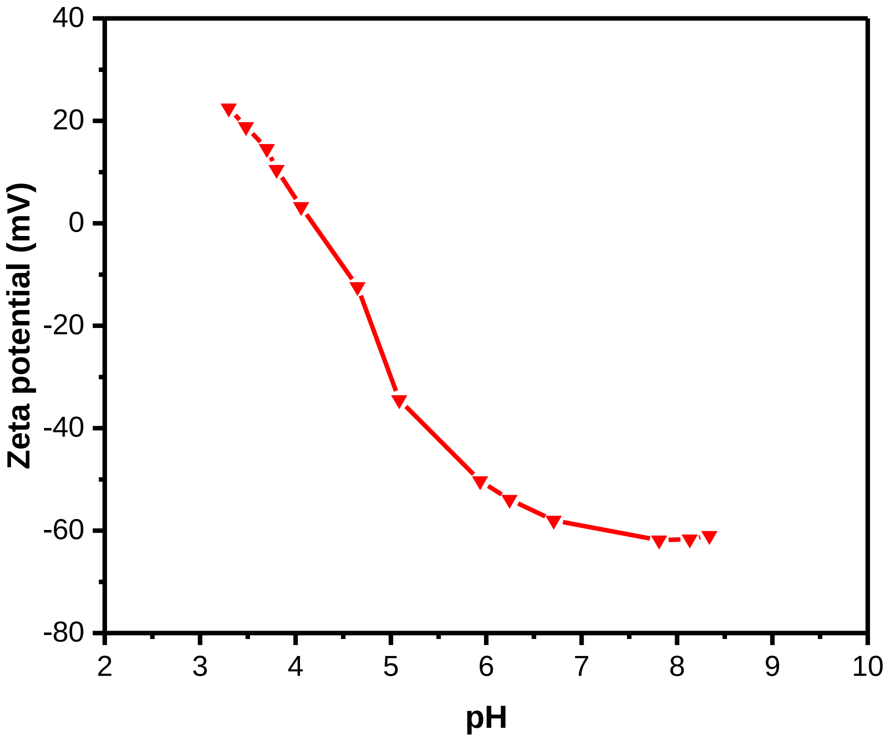

Figure S5. Representative zeta potential of graphene oxide over a range of different pH values.

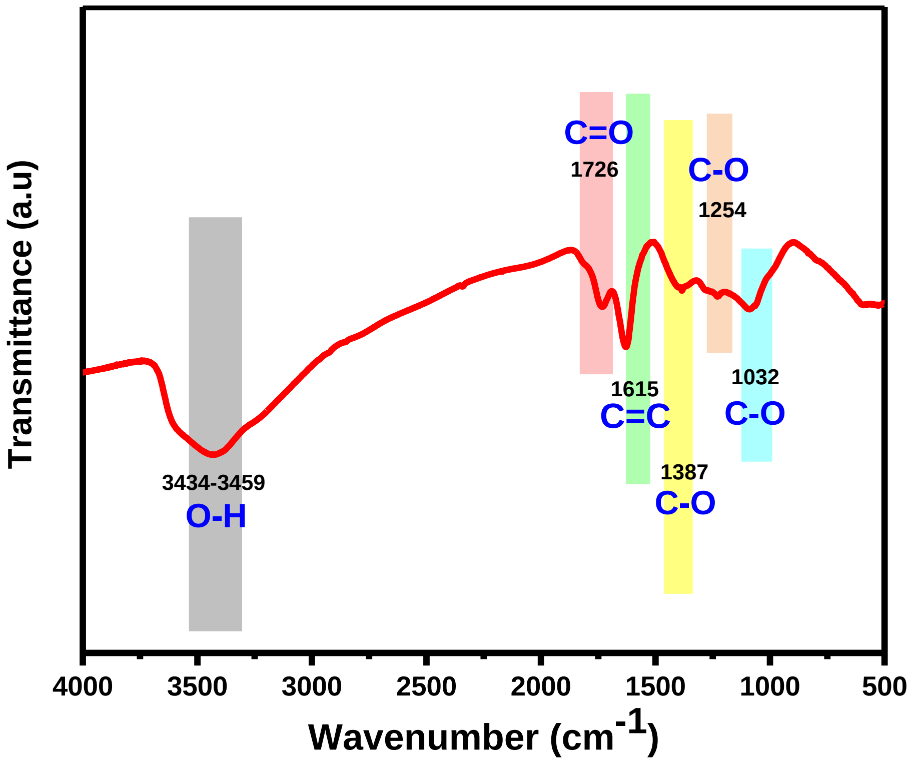

Figure S6. The Fourier transformed infrared (FTIR) spectrum of graphene oxide shows vibrations of functional groups of C–O–C (~1000 cm^{-1}), C–O (1230 cm^{-1}), C=C (~1620 cm^{-1}), C=O (1740–1720 cm^{-1}) bonds and O–H (3600–3300 cm^{-1}).

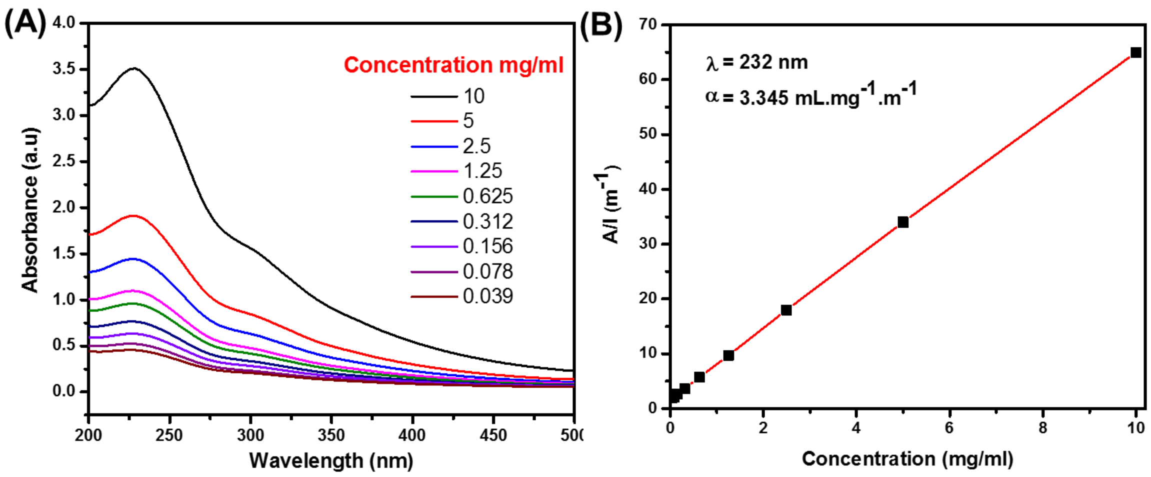

Figure S7. (**A**) UV/Vis absorption spectra of graphene oxide solutions with different concentrations (from 0.039–10 mg/mL) show the main peak around 232 nm. (**B**) The plot of the absorbance (λ = 232 nm) divided by the cell length, versus the concentration. The Lambert–Beer law ($A = \alpha \times C \times l$), allowed the determination of the absorption coefficient (α). This linear relationship fits well with the Lambert-Beer Law, indicating the good water solubility of the GO product.

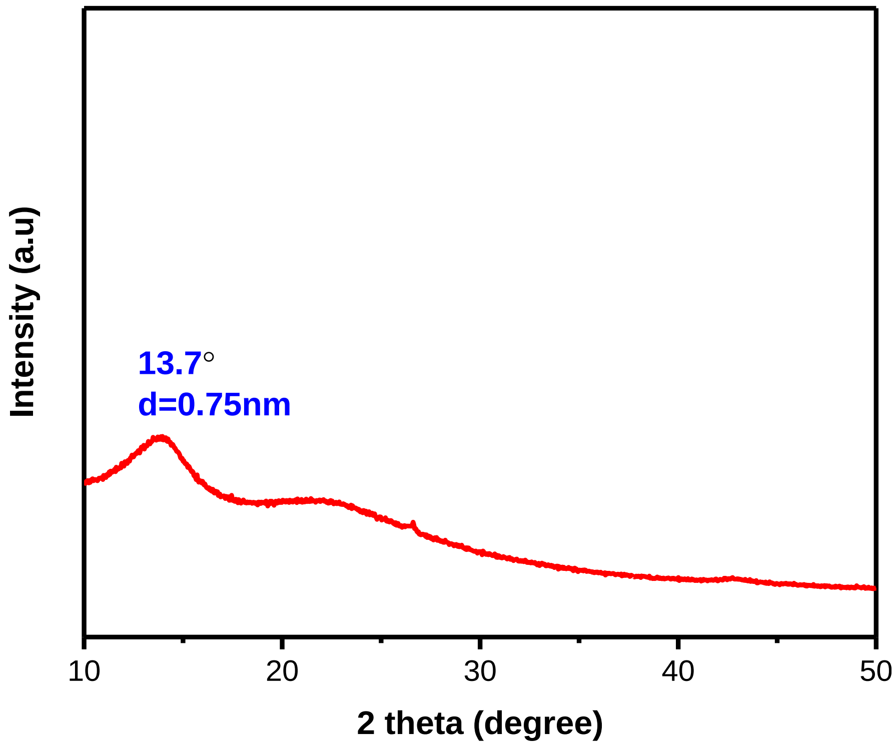

Figure S8. The XRD pattern recorded from graphene oxide shows a (001) peak at 2θ of 13.7°.

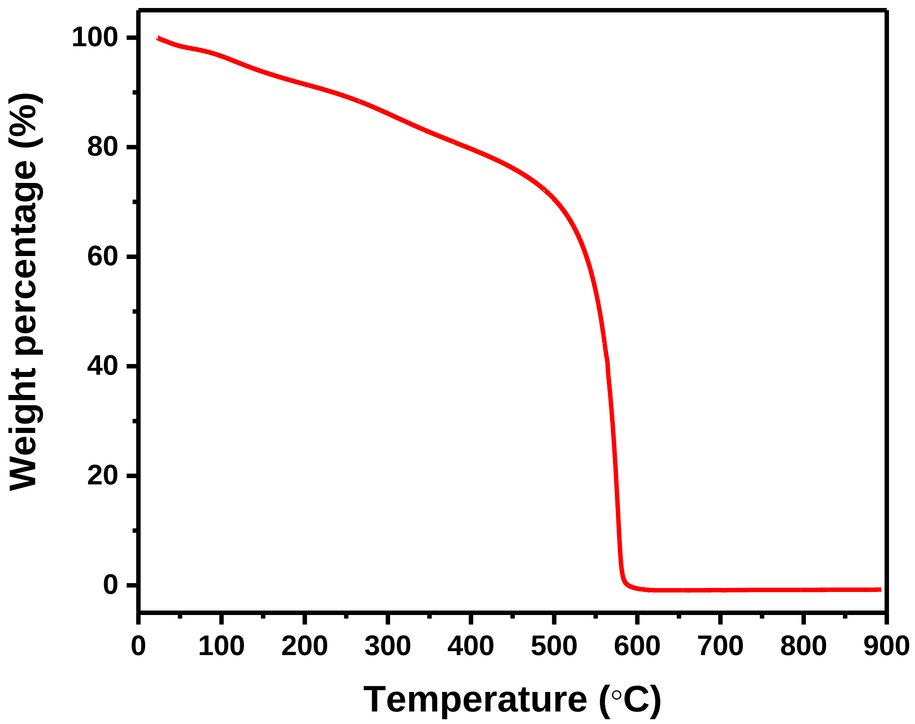

Figure S9. TGA of exfoliated graphene oxide. TGA was performed in the nitrogen atmosphere.

Supplementary Note 2:

Buffers of different pH's were prepared, to investigate the proteolytic activities of CathD and CathL. 21.01 g of citric acid was dissolved in 1 ltr of distilled water and 28.40 g of Na_2HPO_4 in 1 ltr distilled water. Citric acid monohydrate and Na_2HPO_4 buffer solutions were prepared at pH's 3.6, 5, and 7 (as shown below). The final volume of each pH buffer was 50 mL containing freshly prepared 1 mM DTT for CathD (50 ng/mL). The final volume of each pH buffer was 50 mL containing 0.005% Tween 20 (2.5 µL, Sigma) for CathL (50 ng/mL).

pH	Citric Acid Solution (mL)	+	Na_2HPO_4 Solution (mL)
3.6	33.9	+	16.1
5	24.25	+	25.75
7	8.825	+	41.175

Table S1. Characteristic IR bands of the protein linkages.

Approximate Frequency (cm^{-1})	Vibrational Modes	References
1610–1695	CO stretching<br>**C O:** Amide I (C O stretching mode of proteins, α-helix conformation)/C C lipid stretch.	[1]
1480–1575	NH bending and CN stretching<br>**CH_2:** Asymmetric CH_3 bending and CH_2 scissoring, which are linked with elastin, phospholipids and collagen.<br>**C N H:** Absorption of amide II, predominately β-sheet; principally an N H bending coupled to a C N stretching vibrations, C N H bending or/and C N stretching vibrational modes.	[1–5]
1220–1320	CH stretching and NH bending,<br>**C N H:** (ν(CN), δ(NH) amide III, α-helix collagen, tryptophan; and PO_2 − asymmetric phosphate stretching vibrations associated with the phosphodiester groups of nucleic acids.<br>**C N H:** Symmetric stretch: Amide III and CH_3/CH_2 twisting, and/or bending mode of collagens and lipids.	[1–5]
625–765	OCN bending, mixed with other modes of amide II and III	[4,6]
640–800	Out-of-plane NH bending	[4,6]
535–605	Out-of-plane CO bending	[4,6]

Table 2. Kinetic parameters obtained for CathD and CathL for GO using an intraparticle diffusion model.

Adsorbent	Enzyme	k_1 mg/g/min	C *mg/g*	y	R^2
GO (50 µg/mL)	CathD	$k_1 = 0.14$ min^{-1}	0.034939	$0.00732 \pm 4.0427 \times 10^{-4}$	0.9939
	CathL	$k_2 = 3.57 \times 10^{-6}$ g mg^{-1} min^{-1}	2×10^4	0.00866 ± 0.00126	0.9777
GO (500 µg/mL)	CathD	$k_1 = 0.06$ min^{-1}	0.494343	0.00866 ± 0.00126	0.9591
	CathL	$k_2 = 8.0 \times 10^{-7}$ g mg^{-1} min^{-1}	5×10^4	2E-05x + 0.0005	0.9747
GO (1000 µg/mL)	CathD	$k_1 = 0.067$ min^{-1}	0.998941	0.00708 ± 0.00243	0.8996
	CathL	$k_2 = 1.3 \times 10^{-5}$ g mg^{-1} min^{-1}	5×10^4	2E-05x + 0.0003	0.9747

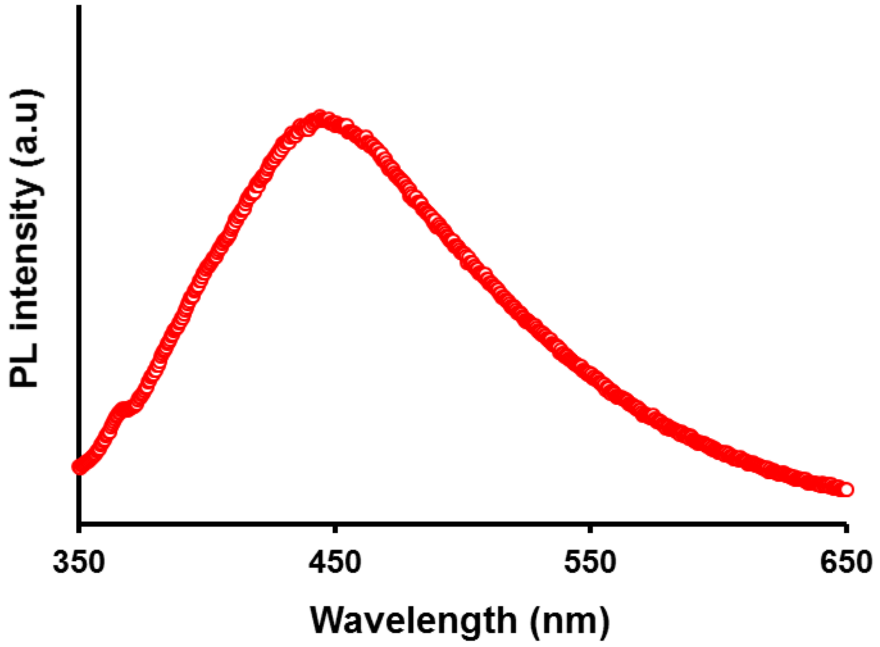

Figure S10. Photoluminescence emission spectrum of graphene oxide.

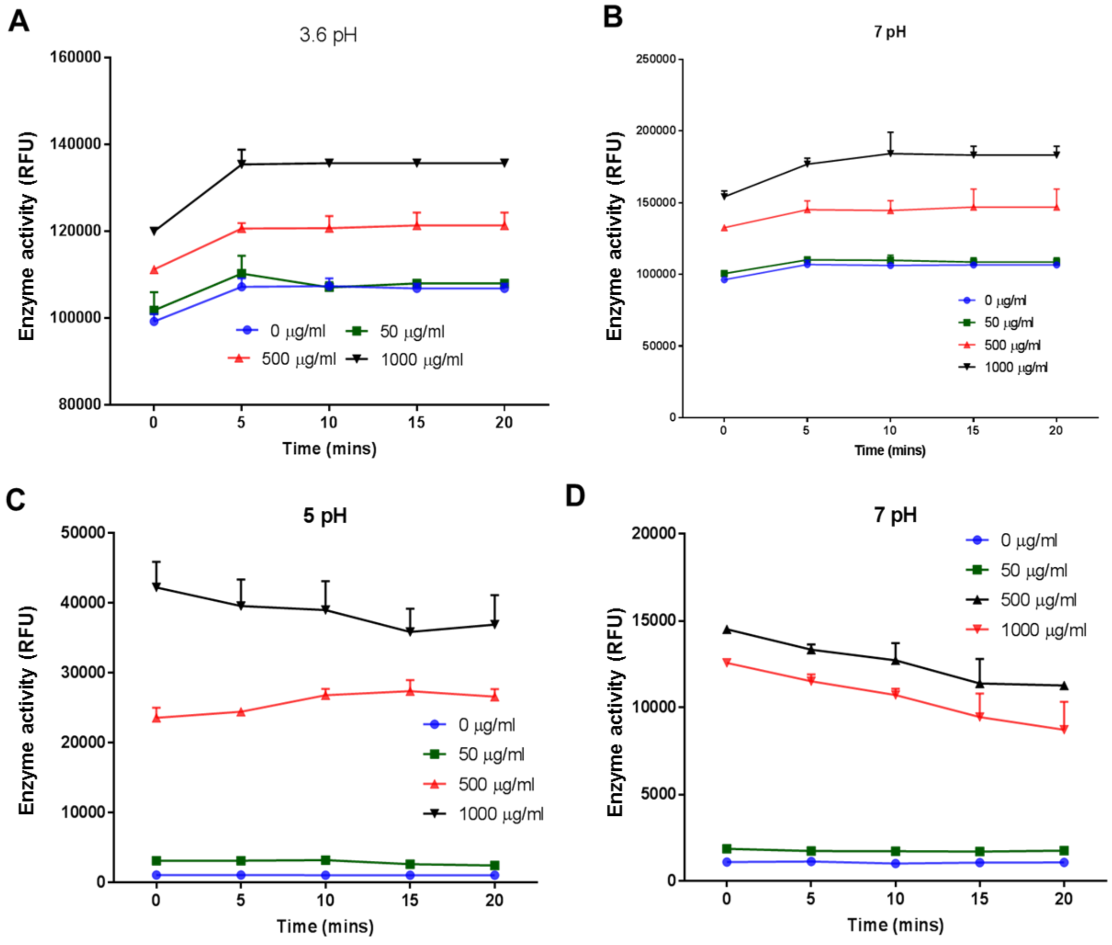

Figure S11. Effect of different concentrations of GO on CathD and CathL fluorescence activities. GO at different concentrations (50, 500, and 1000 µg/mL) were incubated with CathD (**A**, **B**) and CathL

(**C**, **D**) in 96 well plates at different time-points (2, 5, 10, 15, and 20 min) as shown where RFU is relative fluorescence units. Fluorescence signals were determined using a plate reader at Ex/Em: 355/450 nm. Each data point represents the mean of $n = 4$ experiments. Bars show SDs.

References

1. Rehman, S.; Movasaghi, Z.; Darr, J.A.; Rehman, I.U. Fourier transform infrared spectroscopic analysis of breast cancer tissues; identifying differences between normal breast, invasive ductal carcinoma, and ductal carcinoma in situ of the breast. *Appl. Spectrosc. Rev.* **2010**, *45*, 355–368.
2. Staniszewska, E.; Malek, K.; Baranska, M. Rapid approach to analyze biochemical variation in rat organs by ATR FTIR spectroscopy. *Spectrochim. Acta A Mol. Biomol. Spectrosc.* **2014**, *118*, 981–986.
3. Kumar, S.; Barth, A. Following enzyme activity with infrared spectroscopy. *Sensors* **2010**, *10*, 2626–2637.
4. Morhardt, C.; Ketterer, B.; Heißler, S.; Franzreb, M. Direct quantification of immobilized enzymes by means of FTIR ATR spectroscopy–a process analytics tool for biotransformations applying non-porous magnetic enzyme carriers. *J. Mol. Catal. B* **2014**, *107*, 55–63.
5. Barth, A. Infrared spectroscopy of proteins. *Biochim. Biophys. Acta* **2007**, *1767*, 1073–1101.
6. Movasaghi, Z.; Rehman, S.; ur Rehman, D.I. Fourier transform infrared (FTIR) spectroscopy of biological tissues. *Appl. Spectrosc. Rev.* **2008**, *43*, 134–179.

© 2019 by the authors. Submitted for possible open access publication under the terms and conditions of the Creative Commons Attribution (CC BY) license (http://creativecommons.org/licenses/by/4.0/).
